# HERMEES: A Holistic Evaluation and Ranking Model for Energy-Efficient Systems Applied to Selecting Optimal Lightweight Cryptographic and Topology Construction Protocols in Wireless Sensor Networks

**DOI:** 10.3390/s25092732

**Published:** 2025-04-25

**Authors:** Petar Prvulovic, Nemanja Radosavljevic, Djordje Babic, Dejan Drajic

**Affiliations:** 1 School of Computing, Union University in Belgrade, 11000 Belgrade, Serbia; nradosavljevic@raf.rs (N.R.); djbabic@raf.rs (D.B.); 2 School of Electrical Engineering, University of Belgrade, 11000 Belgrade, Serbia; dejan.drajic@etf.bg.ac.rs; 3 Innovation Center of the School of Electrical Engineering in Belgrade, 11000 Belgrade, Serbia

**Keywords:** HERMEES, lightweight cryptography, multi-criteria decision-making model, topology construction protocol, wireless sensor network

## Abstract

This paper presents HERMEES—Holistic Evaluation and Ranking Model for Energy Efficient Systems. HERMEES is based on a multi-criteria decision-making (MCDM) model designed to select the optimal combination of lightweight cryptography (LWC) and topology construction protocol (TCP) algorithms for wireless sensor networks (WSNs) based on user-defined scenarios. The proposed model is evaluated using a scenario based on a medium-sized agricultural field. The Simple Additive Weighting (SAW) method is used to assign scores to the candidate algorithm pairs by weighting the scenario-specific criteria according to their significance in the decision-making process. To further refine the selection, mean shift clustering is utilized to group and identify the highest scored candidates. The resulting model is versatile and adaptable, enabling WSNs to be configured according to specific operational needs. The provided pseudocode elucidates the model workflow and aids in an effective implementation. The presented model establishes a solid foundation for the development of guided self-configuring context-aware WSNs capable of dynamically adapting to a wide range of application requirements.

## 1. Introduction

Wireless Sensor Networks (WSNs) play a vital role in agriculture by collecting crucial data such as soil humidity, temperature, pH, and nutrient levels. These data enable informed decisions to be made regarding the precise levels of irrigation and fertilization necessary for optimal crop growth [1]. A typical WSN consists of multiple interconnected sensor nodes, which enable data collection and transmission to a remote server, where data are stored and processed. Each node consists of a microcontroller, transceiver (RF module), external memory, a power source, and one or more sensors.

The implementation of WSNs presents several challenges, particularly regarding data security and energy consumption. Security is maintained through cryptographic algorithms, ensuring secure transmission of encrypted messages across the network. Energy consumption profoundly impacts the operational lifespan of battery-powered WSN nodes, while encryption algorithms can be computationally intensive and contribute significantly to energy consumption [1].

Lightweight cryptography (LWC) has emerged as a response to these constraints, providing cryptographic primitives specifically designed to be efficient in terms of both computation and memory, while maintaining an acceptable level of security for typical IoT and WSN applications. These algorithms are evaluated not only by their cryptographic strength but also by metrics such as cycles per byte, energy per bit, and implementation cost on microcontrollers or FPGA platforms, allowing more informed decisions in resource-constrained deployments.

Meanwhile, WSN topology construction protocols (TCPs) establish efficient routing of data, minimizing the number of intermediary nodes and balancing network load through activation and sleeping of nodes. A critical aspect of these protocols is their ability to dynamically manage node activity through sleep/wake cycles, ensuring that only a subset of nodes remains active at any time while preserving connectivity and coverage. The challenge is how to maintain a balance between energy savings and the functional requirements of the application, particularly in scenarios requiring high reliability or full spatial coverage of the monitored area. However, the adoption of cryptographic solutions in WSNs is constrained by the limited resources of sensor nodes. A key challenge lies in balancing the trade-offs between the robustness of the algorithm—its resistance to various forms of cryptanalysis and side-channel attacks—and its impact on the system in terms of power consumption, memory usage, execution time, and even physical implementation constraints such as silicon area in hardware-based solutions. More robust algorithms tend to be more demanding, which may compromise the energy efficiency and real-time responsiveness of the network.

Together, the selection of suitable LWC algorithms and TCPs becomes a multi-criteria optimization problem, where the interplay between security, energy, coverage, latency, and device capabilities must be holistically evaluated to ensure sustainable and secure operation of the WSN in real-world conditions.

This paper introduces HERMEES—Holistic Evaluation and Ranking Model for Energy-Efficient Systems. HERMEES provides a simple, joint scoring model to determine the best combination of an encryption algorithm and a topology construction protocol for a given WSN scenario. It applies a multi-criteria decision-making (MCDM) approach using the Simple Additive Weighting (SAW) method for scoring the candidate pairs of LWC and TCP algorithms, followed by mean shift clustering to highlight the best-scored candidate pairs.

The proposed model allows for efficient and simple selection of LWC and TCP algorithms in a WSN, depending on the WSN deployment scenario. Here, we focus on the class of LWC symmetrical algorithms based on the Substitution-Permutation Network (SPN) and the following set of cryptographic algorithms: AES, PRESENT, NOEKEON, LED, and PRINCE. Selected TCPs included in this study are A3, A3 coverage, CDS rule K, and EECDS. The proposed model is evaluated on the deployment scenario of a medium-sized agricultural field, defined by a selected set of relevant LWC and TCP parameters with assigned weights and preferences to lower or higher values. The result is a ranked and clustered list of the most suitable (LWC, TCP) pairs. The model allows flexibility in parameter selection and weight and preference assignment, enabling adaptation to various WSN deployment scenarios.

Although currently demonstrated in a single scenario, HERMEES offers a foundation that could be extended into a broader framework. By compiling a structured repository of parameter values for cryptographic algorithms and topology protocols, along with a set of representative WSN deployment scenarios defined through relevant parameters and weights, the model opens possibilities for future scalability. This approach suggests a path toward guided, self-configuring, context-aware WSN setups, where a user-defined scenario may be used to recommend a suitable configuration from available algorithm and protocol options.

The paper is structured as follows: Section 2 covers the current state-of-the-art and previous work on the LWC algorithms and Topology construction protocols, their properties, and their effects on WSN. Section 3 introduces the proposed method, briefly explains the five phases of the process, compiles the entire process into a pseudocode, and addresses the generic parts of the process. Section 4 explains the simulated scenario used as an example for model evaluation and presents the relevant LWC and TCP features and their values for selected LWC algorithms and topology construction protocols and how the data are prepared for the method. Section 5 presents the results of applying the proposed method to the selected scenario, examines the results, and discusses their validity. Finally, Section 6 offers a discussion of the results, highlighting the possible applications and future directions.

## 2. Related Work

In recent years, the proliferation of Internet of Things (IoT) devices has highlighted the critical need for robust security measures to ensure data integrity, confidentiality, and access control within IoT networks. Cryptography is essential for achieving these security objectives. However, traditional cryptographic protocols often prove inadequate in the resource-constrained environments typical of IoT networks, prompting the development of lightweight cryptographic algorithms and protocols tailored to these specific requirements. In our previous work [2,3,4,5,6,7], we have extensively studied the properties of the lightweight cryptography class of algorithms independently from topology construction protocols. However, we argue that a comprehensive evaluation of these aspects together provides a deeper understanding of the system. Although these issues could be regarded as distinct concerns within their respective layers of abstraction, we consider that examining them together yields more insightful outcomes.

IoT systems are applied across various fields. The scenario discussed in this paper is in the field of agriculture, since IoT/WSN is incredibly important for this sector because it enables smart farming, i.e., using technology to improve efficiency, productivity, and sustainability. In [8], the authors present a case of application of WSN in agriculture, emphasizing that energy efficiency is crucial for WSN sustainability, with data transmission identified as a major factor of energy consumption. Cryptographic algorithms impose additional demands on energy and computational resources, as the traffic needs encryption. Both lightweight cryptography and topology construction protocols are well-researched areas, attracting ongoing academic interest.

Thakor et al. [9] explored the role of lightweight cryptography in mitigating cybersecurity threats in IoT, particularly within industrial control systems. Their performance comparison of lightweight block ciphers highlights the importance of balancing cost, performance, and security in designing cryptographic algorithms for resource-constrained IoT devices. They also identify future research directions to enhance the effectiveness of lightweight cryptographic solutions in various IoT applications. Sivagurunathan et al. [10] provide an in-depth analysis of lightweight cryptographic algorithms specifically designed for Industrial IoT (IIoT). The authors review metrics such as Substitution Permutation Network (SPN) and Feistel Network (FN), highlighting the challenges and opportunities in securing IIoT systems with limited memory, energy, and processing capabilities. Their study emphasizes the importance of standardized lightweight cryptographic algorithms to meet the unique security demands of IIoT.

LWC algorithms share common attributes that can be measured and compared, such as implementation complexity, energy consumption, encryption throughput, latency, and key length. Rana et al. [11] offer a comprehensive overview of the evolving landscape of lightweight cryptographic protocols for IoT networks. Their survey underscores the growing necessity for lightweight cryptography in IoT. The authors conduct a comparative analysis of popular lightweight ciphers and their suitability and security implications for IoT applications. Radhakrishnan et al. [12] evaluate the performance of three lightweight encryption algorithms. Evaluation is based on algorithm performance, security, and suitability to resource-constrained IoT devices, considering factors such as code size and RAM consumption. Panahi et al. [13] evaluate the performance of ten lightweight encryption algorithms, focusing on their applicability in resource-constrained IoT scenarios. The authors assess factors such as memory usage, energy consumption, throughput, and execution time for encryption and decryption operations, offering valuable insights for optimizing network security in IoT. Hatzivasilis et al. [14] present a comprehensive survey of lightweight cryptographic algorithms, focusing on symmetric-key block ciphers for embedded systems. Their evaluation of various block ciphers based on security, performance, and cost provides a nuanced understanding of the suitability of these algorithms across different embedded device architectures, suggesting potential avenues for future research in lightweight cryptography. Banik et al. [15] examine the energy efficiency of lightweight block ciphers, highlighting the significance of energy consumption per encryption operation in resource-constrained environments. Using a model based on CMOS gate energy consumption, they predict optimal round values for energy-efficient block cipher implementations, offering insights into the physical and algorithmic factors influencing energy efficiency in lightweight cryptographic solutions.

Novel results can be seen in the works of Yogaraja et al. [16] and Roy et al. [17], where authors introduce new and improved encryption algorithms with specific concern for devices with limited resources employed in WSN for IoT. Tawalbeh et al. [18] address the growing need for lightweight cryptographic algorithms in mobile and web applications, addressing the challenges posed by the proliferation of smart IoT devices with limited hardware. Their proposed algorithm offers a promising approach to balancing security needs with the inherent resource constraints of IoT environments.

In WSN routing with a focus on energy efficiency and power consumption, novel results are given in Manoharan et al. [19], Mercy et al. [20], and Remesh Babu et al. [21], where authors introduce topology construction protocols designed to minimize energy demands during route creation. Mercy et al. [20] highlight that the primary metrics for topology construction protocols are energy consumption and WSN longevity, noting that these factors also influence overall network security. They propose enhancing security by incorporating trust metrics in topology creation and node selection. Remesh Babu et al. [21] present a topology construction protocol that prioritizes data reliability while focusing on energy consumption at the individual node level. The complexity of a topology construction algorithm, which must be executed on resource-limited WSN devices, is also a critical consideration. The parameters identified in these studies can be integrated into the model proposed in this paper, provided that the compiled data are available for all the candidates.

The lack of a combined assessment of topology construction protocols and lightweight cryptographic algorithms represents a notable gap in current literature. The initial work addressing this topic is by Radosavljević et al. [7], who introduce an MCDM model based on PROMETHEE II for evaluating these components in WSNs, with power consumption as the primary criterion. Their model considers 11 parameters related to LWC and TCP algorithms, providing insight into their impact on network lifetime. A conceptually related approach is presented by Ilieva and Yankova [22], whose model focuses on the evaluation of IoT platform providers, particularly in the agricultural domain, and builds on the work by Ullah et al. [23], where 21 key factors for IoT platform selection are identified, such as scalability, edge computing support, protocol availability, data ownership, and pricing models. While both approaches rely on expert-driven evaluation and share some parameters, such as bandwidth, stability, and security, they address different layers of the IoT architecture. This reflects the broader context of IoT as a layered system that may or may not include WSNs. While WSNs can serve as the data-gathering layer within IoT, they can also operate independently in offline or local deployments. In contrast, many IoT systems transmit data using existing infrastructure like GSM or Wi-Fi, without involving structured sensor networks and local communication among nodes before data reach external systems. These works operate at the system level, focusing on platform capabilities and service offerings. In contrast, our work focuses on algorithm selection at the WSN node level. It considers implementation-level parameters relevant to performance on resource-constrained devices, bridging the gap between network protocol design and embedded system capabilities.

Due to these architectural and functional distinctions, direct comparisons are quite limited, which leaves little existing work to serve as a baseline. Although the methodological foundations are similar, the evaluation targets differ: their model selects service-level IoT solutions, while our model focuses on evaluating node-level implementations within WSNs.

The proposed approach addresses this gap by introducing a flexible, scenario-driven evaluation framework tailored to WSN applications. It supports dynamic parameter selection, allowing evaluations to reflect specific, scenario-driven, deployment contexts, and priorities. By defining deployment scenarios with relevant parameters and assigned weights, our model allows dynamic parameter selection and facilitates informed decision-making tailored to specific WSN applications. Additionally, the model sets the stage for establishing structured repositories: one for algorithm and protocol parameter values and another for typical WSN deployment scenarios, thus enabling scalable, adaptable, and context-aware system design.

## 3. HERMEES

HERMEES requires a clear understanding of the sensor network’s objectives and priorities. The model consists of five phases. In Phase 1, the WSN deployment scenario is formulated to accurately define the scenario. Phase 2 involves the selection of topology construction protocols and assigning values of their parameters. Phase 3 involves the selection of cryptographic algorithms and assigning values of their parameters. Phases 2 and 3 can be conducted in parallel, independently of each other. In Phase 4, weight factors are assigned to parameters, as defined by the scenario, and scores are calculated for each pair of LWC and TCP candidate algorithms. In Phase 5, the scored candidates are ranked and clustered and the best candidates are selected.

**Figure 1:**
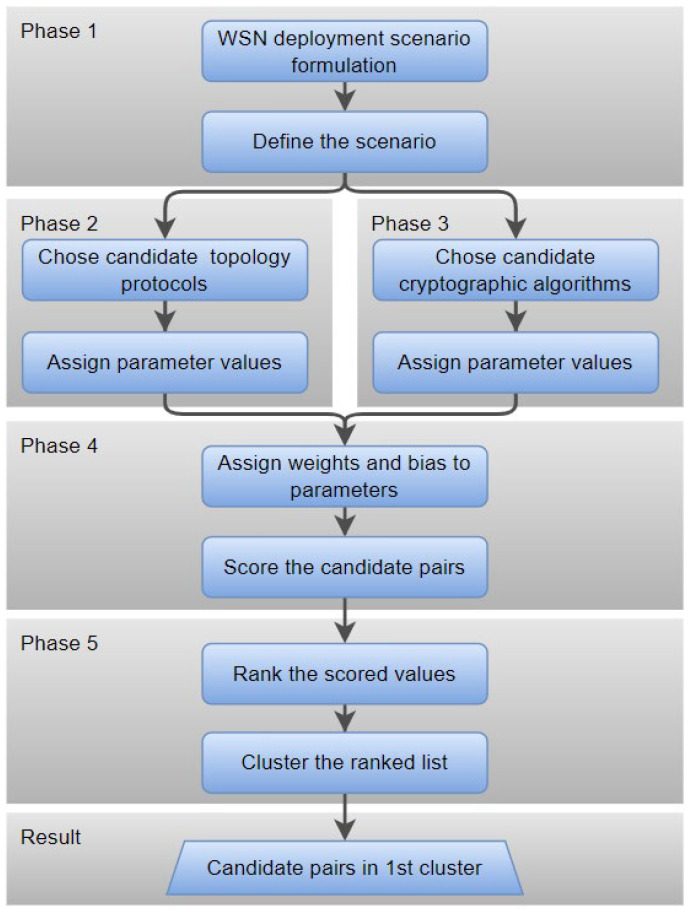
Joint scoring model workflow.

The workflow of the model is presented in Figure 1. Phases are introduced and MCDM, simulation setup, and simulation results are further explained in the following chapters. It should be noted that aside from SPN and TCP parameter values, the scenario itself and available components for the WSN can limit the candidates in a cut-off value style, excluding some of the possible candidates from consideration.

### 3.1. Phase 1: Defining the Scenario

In the envisioned framework, a scenario is defined as a set of relevant parameters for topology protocols and cryptographic algorithms, each with assigned weights and preferences. Weights indicate the relative importance of parameters within a normalized range [0, 1], while preferences describe whether lower or higher values are favored, expressed as min or max. Ideally, users would either select a predefined scenario from a repository—if such a repository is compiled—or define one manually by specifying parameter sets, weights, and preferences. In this study, a single scenario corresponding to medium-sized agricultural fields is defined. Scenario components are denoted as follows: *Cparams* and *Tparams* represent cryptographic algorithms and topology protocol parameters, *Cweights* and *Tweights* represent parameter weights, and *Cprefs* and *Tprefs* define the parameter preferences. The abbreviations *C* and *T* stand for cryptographic algorithms and topology protocols, respectively.

### 3.2. Phase 2: Selection of Topology Construction Protocols

Phase 2 involves selecting topology construction protocols and assigning values to parameters defined by *Tparams*. The primary objective of the topology construction protocol is to optimize both the message count and the coverage of the observed area. In general, selection depends on the implementations available for the equipment used for WSN deployment. The output of this phase includes sets of selected protocols and their corresponding parameter values, denoted as *Tcandidates* and *Tparamvalues*, respectively.

### 3.3. Phase 3: Selection of Cryptographic Algorithms

Phase 3 involves selecting cryptographic algorithms and assigning values to parameters defined by *Cparams*. In general cases, selection can be made by considering algorithms available and that are feasible on the equipment used for WSN deployment, considering that some algorithms might have hardware requirements that are unavailable on the selected devices. The aim is to select algorithms that achieve a balance between security, energy efficiency, and network performance, which is crucial for the effectiveness of a WSN. The output of this phase includes sets of selected algorithms and their significant parameters. Sets are named *Ccandidates* and *Cparamvalues*, respectively.

### 3.4. Phase 4: Scoring

Phase 4 applies the Simple Additive Weighting (SAW) method [24] to calculate scores for each pair of cryptographic algorithm and topology protocol candidates. SAW is particularly effective when criteria importance is well-defined, making it suitable for decision-making scenarios such as supplier selection, product choice, or investment evaluation.

The first step involves assigning weights and preferences to the selected parameters of cryptographic algorithms and topology protocols. Weights reflect parameter importance, expressed as values between 0 and 1, with the total sum equal to one. Preference values indicate whether lower or higher parameter values are preferred, represented as *min* and *max*. These values are defined in Phase 1 within the sets *Tweights, Cweights, Tpref*, and *Cpref*. SAW coefficient candidate parameters are defined in a dictionary named *saw*, indexed by the parameter names of both *Tweights* and *Cweights*. Preference values are similarly stored in a dictionary named *pref*, indexed by parameter names from *Tpref* and *Cpref*.

The second step of Phase 4 calculates scores for all pairs of cryptographic algorithms and topology protocols. The score *S* for each pair is calculated using SAW weighting coefficients and normalized parameter values. The Max normalization technique [24] is applied as follows:

(1) $$S=\sum_{p\in P}{saw}_{p}\cdot norm(p)$$

where *P* is the set of all selected parameters (union of *Cparams* and *Tparams*), *saw_p_* is the weight factor of the parameter *p*, and *norm(p)* is the normalized value of the parameter *p*. The normalized value is calculated by dividing the parameter values by the maximum parameter value. Depending on the parameter’s preference, if lower values are preferred, the normalized value is subtracted from 1 to preserve its relative importance in the weighted ranking. Normalization is defined as:

(2) $$norm\left(p_{i}\right)=\left\{\begin{array}{c} \;\;\;\;\;\;\;\;\frac{p_{i}}{p_{max}},\;\;if\;{pref}_{p_{i}}=max\\ 1-\frac{p_{i}}{p_{max}},\;\;if\;{pref}_{p_{i}}=min \end{array}\right.\;\;$$

where *p_i_* is the parameter *p* value of *i*-th candidate, *p_max_* is the maximum value of the parameter *p* in all candidates, and ${pref}_{p_{i}}$ is the assigned preference to larger or smaller value, assigned to the parameter *p_i_*.

The output of Phase 4 is the list containing all pairs of cryptographic algorithm and topology protocol candidates and their calculated scores, defined as a dictionary named *S*. Dictionary *S* contains score values, indexed by the candidate pairs. Scoring can be implemented using Algorithm 1. Function SCORE takes the lists of topology protocol and cryptographic algorithm candidates and their selected parameters, the values of the parameters, SAW coefficients, and parameter preferences as input parameters. To calculate the scores, it is necessary to normalize the parameter values. For this purpose, an auxiliary function NORMALIZE is defined, which takes a matrix of values *M*, where columns represent parameters and rows represent candidates, and a list of parameter preferences *pref* and returns a matrix of normalized values. Normalization is performed column-wise, where the maximum value in a column is identified, and all the values in that column are divided by the maximum. Depending on the preference, normalized value is subtracted from 1, according to (2). Columns in *M* represent the selected parameters and rows represent the candidate algorithms. Elements in *P* represent the preference values assigned to parameters. Subsequently, the score is calculated for each pair of cryptographic algorithms and topology protocols, according to (1), and a dictionary *S* is formed, providing the result of the function and the output of Phase 4.
**Algorithm 1** Scoring**function** NORMALIZE(*M*, *pref*)**for** each row *c* in *M* do**for** each column *p* in *M* do$M[c,p]\leftarrow \left\{\begin{array}{c} \frac{M[c,\;\;p]}{max(M\left[\;{\_}_{,}p\right])},\;\;pref\left[p\right]=max\\ 1-\frac{M[c,\;\;p]}{max(M\left[\;{\_}_{,}p\right])},\;\;pref\left[p\right]=min \end{array}\right.$**end****end****return** *M***end** function**function** SCORE(*Ccandidates, Tcandidates, Cparams, Tparams**Cparamvalues, Tparamvalues, saw, pref*)*Cparamvalues* ← NORMALIZE(*Cparamvalues, pref*)*Tparamvalues* ← NORMALIZE(*Tparamvalues, pref*)*S* ← [ ])**for** each *c* in *Ccandidates* **do****for** each *t* in *Tcandidates* **do***S* [(*c,t*)] ← $\sum_{p\in Cparams}saw\left[p\right]\cdot Cparamvalues\left[c,p\right]$
$+\sum_{p\in Tparams}saw\left[p\right]\cdot Tparamvalues[t,p]$.**end****end****return***S***end function**

### 3.5. Phase 5: Selection of the Best Candidates

Phase 5 focuses on ranking the list of scored pairwise combinations of cryptographic algorithms and topology protocols and selecting the most suitable candidates. The scores obtained in Phase 4 are used to form a final ranking, where a higher score indicates a more appropriate combination for the given scenario. While selecting only the highest scoring candidate might be the most straightforward solution, this approach may overlook other comparable options, especially when the score differences are small.

To mitigate this, the model selects a group of candidates that stand out at the top of the sorted list of scores. The mean shift clustering algorithm [25] is applied to the sorted list, grouping candidates based on their scores. Candidate pairs from the first cluster are presented as the best candidates for the given scenario. Mean shift was chosen primarily for its simplicity of use and minimal need for parameter tuning. It does not require prior knowledge of the number of clusters, which is beneficial in cases where the structure of the data is not known in advance. In this application, the bandwidth parameter is derived directly from the number of candidates, making the method easy to integrate without extensive calibration. Mean shift avoids assumptions about the number or shape of clusters and naturally identifies dense regions in the score distribution. These characteristics make it a suitable and practical choice for selecting a subset of high-ranking candidates in an unsupervised and data-driven way.

Ranking is implemented using Algorithm 2. The auxiliary function JOINTRANK accepts a list of scored pairs. First, the list is sorted using the auxiliary function SORT, which sorts a list of dictionaries by values while maintaining the indices associated with the values. The sorted list is then processed using the mean shift clustering algorithm [25]. The algorithm is first initialized and then executed on the list of scored candidate pairs, which produces a list of labels assigned to candidate pairs, indexed respectively. As the candidate pairs are sorted in descending order, the first cluster contains the candidate pairs with the same assigned label at the start of the list. The resulting first cluster is extracted and returned as the output of Phase 5.
**Algorithm 2** Ranking**function** JOINTRANK(*scoredPairs*)*rankedPairs* ← SORT(*scoredPairs*)*meanShift* ← sklearn.cluster.MeanShift(*bandwidth* = $\frac{1}{len(rankedPairs)}$)
*meanShift*.fit(*rankedPairs*)*rankedPairsLabels* ← *meanShift*.*labels_**firstCluster.* ← [ ]**for** *i* **in** range(len(*rankedPairs*))**if** *rankedPairsLabels[i]* = *rankedPairsLabels [0]**firstCluster.*append(*rankedPairs[i]*)**end****end****return** *firstCluster***end function**

### 3.6. HERMEES Algorithm

Considering the previously defined phases and their implementation, the pseudocode listed in Algorithm 3 illustrates the implementation of the entire process. Here, PHASE1(*ds*) represents the process of defining a scenario by selecting from a repository or defining a specific case, while PHASE2(*Tparams, ds*) and PHASE3(*Cparams, ds*) represent phases 2 and 3, respectively, where candidate selection is performed based on the scenario and parameter values are defined based on selected parameters.
**Algorithm 3** HERMEES algorithm pseudocode**input:** WSN deployment scenario *ds***output:** selected candidates*(Cparams, Tparams, Cweights, Tweights, Cbias, Tbias)* ← PHASE1(*ds*) ▷ PHASE 1*Tcandidates, Tparamvalues* ← PHASE2(*Tparams, ds*)          ▷ PHASE 2*Ccandidates, Cparamvalues* ← PHASE3(*Cparams, ds*)        ▷ PHASE 3*saw* ← *Cweights* + *Tweights*                     ▷ PHASE 4*bias* ← *Cbias* + *Tbias**scoredCandidates* ← SCORE(*Ccandidates, Tcandidates, Cparams, Tparams*            *Cparamvalues, Tparamvalues, saw, bias*)*selectedCandidates* ← JOINTRANK(*scoredCandidates*)         ▷ PHASE 5**return** *selectedCandidates*

## 4. WSN Deployment Scenario Parameters

HERMEES is evaluated on a deployment scenario representing a medium-sized agricultural field. This scenario is defined by a selected set of relevant LWC and TCP parameters with assigned preferences and weights that reflect key performance and resource constraints in WSNs. These parameters include: Coverage coefficient, Overcoverage coefficient, Total message count, Flow rate, Gate equivalent, Delay per round, and Energy consumption. The selection and weighting of these parameters are grounded in both technical considerations and expert consensus, obtained through a questionnaire-based study presented in [7], where experts evaluated the importance of various metrics specifically for the agricultural field scenario, and the resulting insights guided the parameter selection in this paper.

In the evaluated scenario, four topology construction protocols were considered, as provided by the simulation environment: A3, A3 coverage, CDS rule K, and Energy-Efficient Connected Dominating Set (EECDS). Parameter values for these protocols were obtained through simulations conducted under the assessed scenario conditions. For the topology construction protocols, the Coverage Coefficient *(CC)* represents the ratio of the monitored area successfully covered by active sensor nodes. The Overcoverage Coefficient *(OC)* captures redundancy in coverage, quantifying how much overlap exists among nodes covering the same area. These parameters help assess how effectively the network balances spatial efficiency and fault tolerance. The Total Message Count coefficient *(TMC)* represents the communication load induced by each protocol. This metric directly relates to energy consumption and WSN lifespan, as each message must be encrypted before transmission. Some topology algorithms generate more intra-network communication due to their routing strategies and node sleep scheduling. The TMC metric links the impact of topology decisions to the demands placed on cryptographic processing.

For lightweight cryptographic algorithm evaluation, five SPN-based algorithms were selected: AES, PRESENT, NOEKEON, LED, and PRINCE. Key characteristics evaluated include: Energy consumption per bit (*EC*), which quantifies the energy cost of processing one bit of data, in picojoules (pJ); Flow Rate (*FR*), which indicates data throughput, affecting communication speed; Gate Equivalent (GE), which estimates hardware implementation complexity by counting logic gates required and impacts hardware dimensions; and Delay per Round (*DR*), which measures latency introduced per encryption round, in nanoseconds, affecting throughput and latency. These metrics reflect the trade-offs between performance, resource usage, and implementation feasibility on resource-constrained devices.

Altogether, this parameter set supports a comprehensive and scenario-specific evaluation of WSN performance. It is guided both by practical requirements and domain expert input.

### 4.1. Simulation Setup and TCP Parameter Values

The simulation is conducted using the Atarraya simulator [26], an open-source, Java-based tool designed for researching topology construction and WSN protocols. Atarraya allows for customization and modification, though it does not account for encryption methods or the associated energy costs. The simulation modeled the topology of the monitored area, calculating the overall message count coefficient based on the number of neighboring nodes used by the observed node for communication. The scenario emulated typical conditions of mid-sized monoculture farms in Serbia. A consistent set of parameters, detailed in Table 1, is applied across all the topology construction protocols discussed in this paper. It is important to highlight that these simulation parameters significantly influence the performance of the applied TCPs.

The primary metrics used to evaluate the efficiency of the simulated protocols include the number of sent and received packets and the total amount of data transmitted and received, measured in bytes. Additionally, the simulator tracks the coverage ratio of the monitored area, which depends on the communication radius of the cluster head nodes. Since the simulator records only message counts and node positions, we extended the simulation to calculate additional metrics: coverage and overcoverage coefficients, based on node positions and considering overlaps.

**Figure 2:**
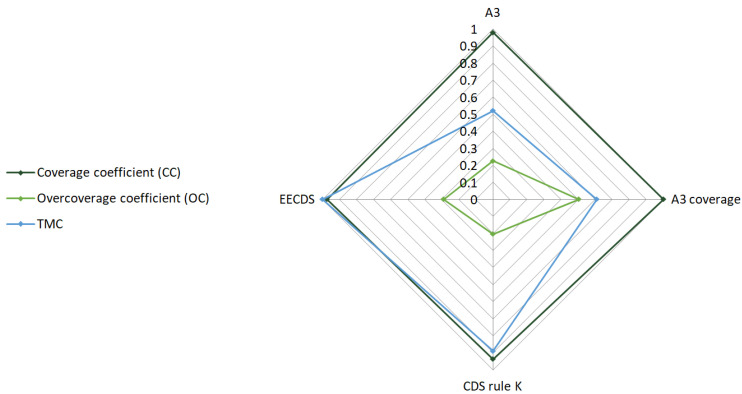
Parameter values for selected topology construction protocols.

The parameters concerning the topology construction protocols include the Coverage Coefficient (*CC*), Overcoverage Coefficient (*OC*), and Total Message Count coefficient (*TMC*). Their interpretation and relevance have been previously discussed. The *TMC* for the i-th candidate protocol is computed by normalizing the total number of messages transmitted and received by both cluster and regular nodes to a [0, 1] range, as defined by the following formula:

(3) $${TMC}_{i}=\frac{{Sch}_{i}+{Rch}_{i}+{Srn}_{i}+{Rrn}_{i}}{{Sch}_{max}+{Rch}_{max}+{Srn}_{max}+{Rrn}_{max}}$$

where *Sch_i_* represents the total data sent by the cluster node, *Rch_i_* signifies the total data received by the cluster node, *Srn_i_* denotes the total data sent by the regular node, *Rrn_i_* represents the total data received by the regular node, and *Sch_max_*, *Rch_max_*, *Srn_max_*, and *Rrn_max_* represent the maximum totals of all the considered topology construction protocols. All values are measured in bytes, and normalization ensures fair comparison between protocols with different communication patterns. The values of these parameters for the selected TCPs are derived from simulation results and presented in Table 2. Figure 2 illustrates the parameter values within the evaluated scenario.

**Figure 3:**
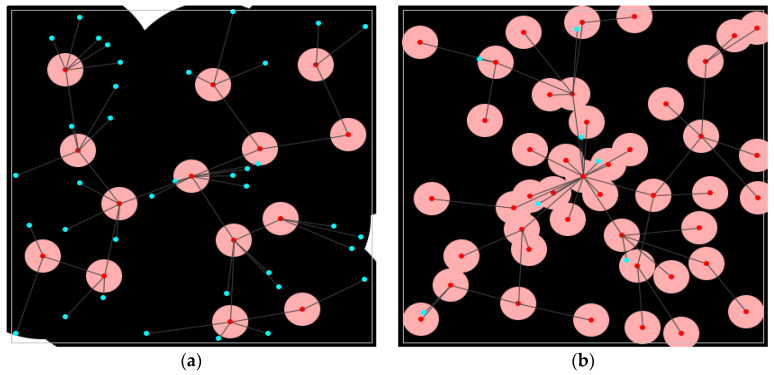

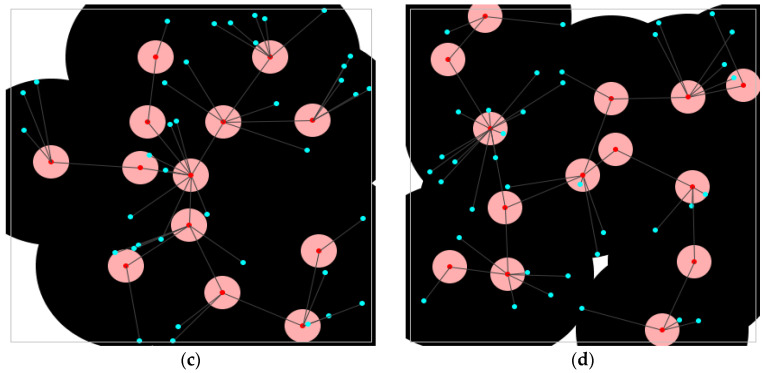
Topology construction protocols node distribution: (**a**) A3 protocol node distribution; (**b**) A3 coverage protocol node distribution; (**c**) CDS rule K protocol node distribution; (**d**) EECDS protocol node distribution.

Graphical representations of all considered topologies show the spatial arrangement of nodes within the observed area, providing a clear overview of how nodes are positioned across the deployment field. These diagrams also indicate the assigned roles of nodes, distinguishing between cluster heads and regular nodes that are involved in the process of data collection. Figure 3 displays the distributions of nodes for each of the selected topology construction protocols (TCPs), along with the corresponding coverage maps that illustrate the area monitored by the deployed network. In these figures, cluster heads are marked with red dots to differentiate them from the regular nodes, which are shown using blue dots.

### 4.2. LWC Parameter Values

**Figure 4:**
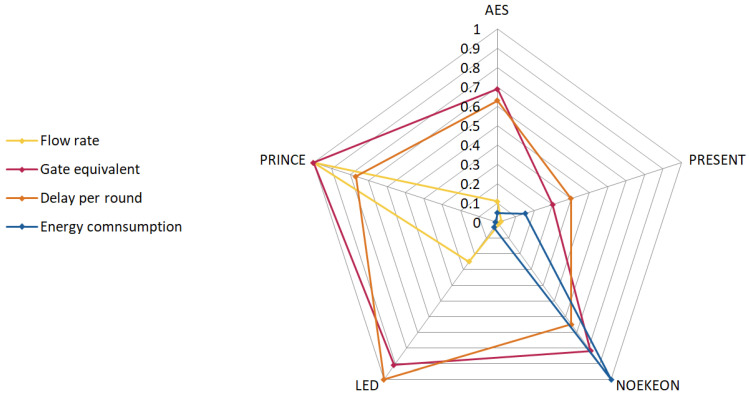
Normalized SPN LWC algorithms characteristics.

For the LWC algorithm parameters, we consider Flow Rate (*FR*), Gate Equivalent (*GE*), Delay per Round (*DPR*) in nanoseconds, and Energy per Byte (*EPB*) in picojoules (pJ). Their roles and relevance within the evaluation have been discussed earlier in this section. The initial and normalized values for these parameters in the selected LWC algorithms are presented in Table 3 and Table 4. Normalized parameter values are illustrated in Figure 4.

### 4.3. Weights and Biases

Table 5 outlines the parameters and their influence on the scoring outcome, indicating whether a maximum or minimum value is preferred, as used in (2).

**Figure 5:**
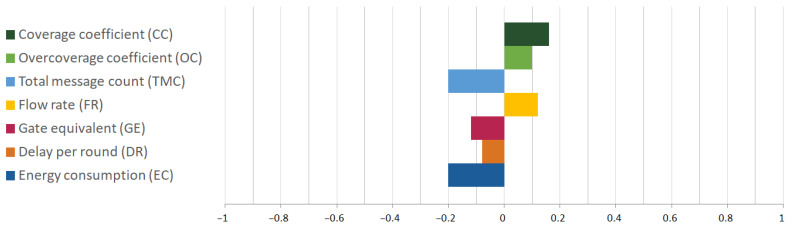
Normalized parameter weight factors, with assigned preference.

Weights are assigned to the parameter values using the SAW method, as defined in (1). Table 6 presents the weighting factors for each parameter, derived through expert analysis specific to the simulated scenario [7]. These weights are normalized using L1 normalization, with the sum equal to 1, as shown in the last column. Figure 5 illustrates normalized parameter values, with assigned preference towards higher or lower values as positive and negative values, respectively.

## 5. Results

**Figure 6:**
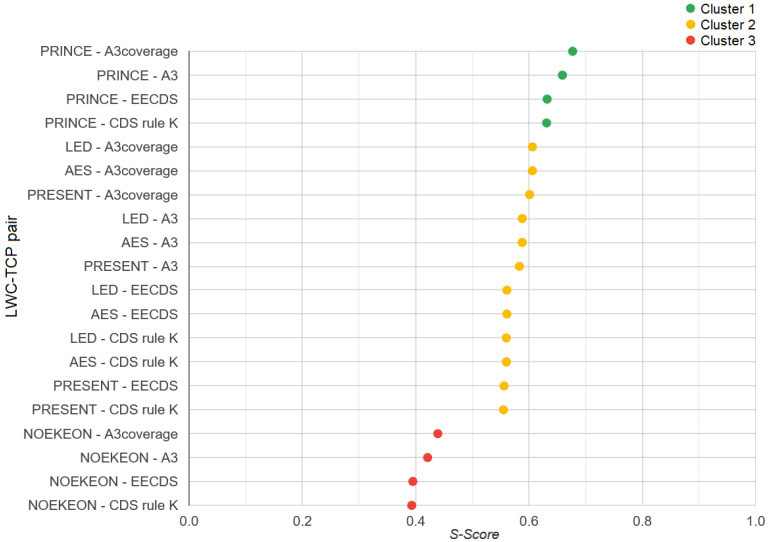
Clustered *S* score values.

The proposed model, HERMEES, was applied to evaluate four selected topology construction protocols, A3, A3 Coverage, CDS Rule K, and EECDS, and five lightweight cryptographic algorithms, AES, LED 128, NOEKEON, PRESENT, and PRINCE. The scoring value is represented by the score *S*, as defined in (1), with higher values indicating better performance. Table 7 shows the *S* values for the combinations of topology construction protocols and LWC algorithms under consideration. In Table 8, these combinations are sorted by their calculated scores, with clusters assigned using mean shift clustering. The results from Table 8 are visually represented in Figure 6. The final result of HERMEES is presented in Table 9, listing the candidates sorted by their *S* score, from the best performing pair for the defined scenario.

## 6. Discussion

The results indicate that for the assessed scenario, the combination of A3coverage and PRINCE received the highest score. Mean shift clustering identified three distinct clusters. In the lowest-ranked cluster, the NOEKEON algorithm stands out with a notably low EC score. This outcome aligns with expectations, as Table 3 shows that NOEKEON has a significantly lower flow rate and higher energy consumption. These factors were weighted heavily by the SAW method, while parameters like gate equivalent and delay per round, where NOEKEON performs moderately well, were assigned lower weights, indicating that they are of lesser importance in the simulated scenario.

In the top-ranked cluster, only the PRINCE LWC algorithm is present, which is also consistent with expectations. As seen in Table 3, PRINCE has a significantly better flow rate and energy consumption than the other candidates. Although PRINCE is the weakest candidate in terms of gate equivalent and delay per round, these parameters are less critical in the simulated scenario, as reflected in their lower SAW weight coefficients. The second cluster contains the remaining three LWC algorithms, each with its strengths and weaknesses. However, none stands out significantly from the others, resulting in close scores with no major deviations to suggest a markedly better candidate. It is also noteworthy that the A3 and A3coverage protocols are more prevalent among the higher-ranked candidates. This is expected, as both protocols have higher coverage and overcoverage coefficients and a lower total message count, as shown in Table 2. This is particularly relevant given that TMC is the parameter with the highest SAW weight in this scenario.

To put the results in perspective, we can make a simple estimation of the power consumption of a single node in the WSN based on the chosen combination of LWC and TCP. We calculate the traffic through a regular node as the sum of the total sent and received data and then multiply it by the energy consumption for processing the traffic through the cryptographic algorithm. Estimates based on data in Table 2 and Table 3 are compiled in Table 10. Energy consumption is expressed in millijoules (mJ).

It is evident that PRINCE provides the best performance, in contrast to NOEKEON, which aligns with the ranked list of candidates. While energy consumption is a crucial factor in the simulated scenario, it is not the only one. Other factors considered in the scenario rank the PRINCE-A3 pair as the second-best option. Although the A3coverage energy consumption estimate is worse than other TCPs, A3coverage has the second-best total message count, which is the TCP parameter with the highest assigned SAW weight coefficient. This demonstrates that the joint scoring model accounts for all parameters, weighing them according to their relative importance, as expressed by the assigned SAW weights, in the defined scenario.

In one of the previous works, an MCDM model based on the PROMETHEE II ranking method was presented [7], which considers 11 parameters and calculates scores through 5 steps. The model described here considers seven parameters and calculates scores in two phases. Both models go through the initial three phases, which involve selecting candidates and parameters and gathering parameter values through simulation. In this respect, the model described here offers simpler implementation while maintaining robustness.

One of the key limitations is the absence of a repository containing parameter values for cryptographic algorithms and topology protocols. In this work, the values were obtained partially through simulation and partially derived from existing research. Developing a standardized repository with parameter values would enable faster model application in various scenarios and reduce reliance on additional simulation or experimental data collection. However, creating this repository requires conducting extensive simulations across various network sizes and environmental conditions, which involves considerable time and computational resources.

By selecting candidate algorithms and protocols, identifying key attributes of cryptographic algorithms and topology protocols, and assigning weights and preferences, the model can be adapted to specific deployment needs. This adaptability allows the approach to address a variety of environments and WSN scenarios. In an ideal implementation, parameter values for cryptographic algorithms could be compiled into a structured repository, based on existing research such as [10]. These parameters are generally determined by the hardware resources available in the WSN, rather than by the deployment scenario. Based on the hardware characteristics and manufacturing technology of the devices used, suitable cryptographic candidates could be selected. Likewise, topology protocol parameters could be simulated for representative deployment scenarios, contributing to the development of a complementary repository that supports broader applicability of the model.

Integrating such parameter databases with MCDM models guided by user input would enable a system capable of scenario-driven, self-configuring, and context-aware WSN setup. This approach would support users with limited technical expertise, where the only required input is a description of the deployment scenario. The system would then determine the optimal configuration based on the scenario characteristics and available algorithms and protocols compatible with the devices in use.

## 7. Summary

This paper introduces **HERMEES**, a model designed to support the selection of an optimal combination of a message encryption algorithm and a topology construction protocol in Wireless Sensor Networks (WSNs). HERMEES is built upon a multi-criteria decision-making (MCDM) approach, with a focus on evaluating lightweight cryptographic (LWC) algorithms and topology construction protocols (TCP) in combination rather than treating them separately. This integrated evaluation provides deeper insights into system performance and enhances decision-making for WSN deployment.

The model builds upon previous work by introducing several key improvements. It emphasizes scenario-driven evaluation, allowing flexible parameter selection based on specific WSN deployment conditions. It also incorporates dynamic parameter inclusion, enabling the model to adapt to different system requirements. Additionally, HERMEES introduces the idea of structured repositories for storing parameter values of LWC and TCP algorithms, along with predefined typical WSN deployment scenarios as a useful direction for future development.

In this study, the model was applied to evaluate four topology construction protocols—A3, A3 Coverage, CDS Rule K, and EECDS—and five lightweight cryptographic algorithms—AES, LED 128, NOEKEON, PRESENT, and PRINCE. Evaluation was based on the *S* score metric, where higher values indicate better performance. Results show that the A3 Coverage-PRINCE combination achieved the highest score, followed by A3-PRINCE. Three clusters were identified: the first cluster was dominated by the PRINCE algorithm, while the last cluster was characterized by the NOEKEON algorithm. These results align with the parameters and weights defined for the selected scenario.

HERMEES provides a straightforward and effective MCDM model that emphasizes evaluating WSN components in combination. The model includes five phases and uses the SAW method for scoring each LWC–TCP pair. Mean shift clustering was applied to group results and highlight strong candidates.

This approach lays the foundation for integrating MCDM models driven by user input into supervised, auto-configurable, context-aware WSNs. Such systems could automatically adapt based on scenario-specific requirements, simplifying the deployment process for non-expert users. These models can be incorporated into WSN systems, allowing nodes to be automatically configured to use the selected algorithms from the available implementations.

Future research will focus on expanding the model’s applicability by compiling a comprehensive repository of topology construction protocol and lightweight cryptographic algorithm parameters, along with a diverse set of curated WSN deployment scenarios. These additions are expected to increase the model’s adaptability across a wider range of real-world conditions. By combining MCDM techniques with scenario-based evaluation and structured data resources, HERMEES aims to support more flexible and secure WSN deployments. We believe that this approach can simplify configuration tasks and help enable smarter, more autonomous sensor networks across diverse application domains.

## Figures and Tables

**Table 1:** Atarraya simulation setup.

Parameter	Value
Area surface (m)	500 × 500
Node communication radius (m)	125
Node sensing radius (m)	25
Distribution	normal distribution
Small packet size (B)	12 to 25
Large packet size (B)	26 to 65

**Table 2:** Parameter values for selected topology construction protocols.

Parameter	A3	A3 Coverage	CDS Rule K	EECDS
Coverage coefficient (*CC*)	0.9787	1	0.9365	0.9721
Overcoverage coefficient (*OC*)	0.2266	0.5054	0.2034	0.2891
Cluster head data—Total sent (*Sch*) (B)	2259	4053	2495	3398
Cluster head data—Total received (*Rch*) (B)	9554	18,320	12,172	16,016
Regular node data—Total sent (*Srn*) (B)	2349	758	4216	4757
Regular node data—Total received (*Rrn*) (B)	9654	4753	21,687	21,436
*TMC*	0.52	0.61	0.89	1

**Table 3:** SPN LWC algorithms characteristics.

Algorithm	100 KHz Flow Rate in kbps (*FR*)	Gate-Equivalent (*GE*)	Delay Per Round in ns (*DR*)	Energy Consumption in μJ/bit (*EC*)
AES	57	2400	3.32	42.38
PRESENT	12.4	1030	2.09	124.56
NOEKEON	3.44	2880	3.41	837.90
LED	133.33	3194	5.25	23.95
PRINCE	533.3	3491	4.06	6.54

**Table 4:** Normalized SPN LWC algorithms characteristics.

Algorithm	*nFR*	*nGE*	*nDR*	*nEC*
AES	0.11	0.69	0.63	0.05
PRESENT	0.02	0.30	0.40	0.15
NOEKEON	0.01	0.82	0.65	1
LED	0.25	0.91	1	0.03
PRINCE	1	1	0.77	0.01

**Table 5:** Parameter preferences.

Parameter	Bias
Coverage coefficient (*CC*)	max
Overcoverage coefficient (*OC*)	max
Total message count (*TMC*)	min
Flow rate (*FR*)	max
Gate equivalent (*GE*)	min
Delay per round (*DR*)	min
Energy consumption (*EC*)	min

**Table 6:** Parameter weight factors.

Parameter Weight Factor	SAW Value (0–1 Range)	L1 Normalized Values
Coverage coefficient (sCC)	0.8	0.16
Overcoverage coefficient (sOC)	0.5	0.10
Total message count (sTMC)	1	0.20
Flow rate (sFR)	0.6	0.12
Gate-equivalent (sGE)	0.6	0.12
Delay per round in ns (sDR)	0.4	0.08
Energy consumption per byte in pJ (sEC)	1	0.20

**Table 7:** Scores of pairs of considered TCP and LWC.

	A3coverage	A3	EECDS	CDS Rule K
**PRINCE**	0.68	0.66	0.63	0.63
**LED**	0.61	0.59	0.56	0.56
**AES**	0.61	0.59	0.56	0.56
**PRESENT**	0.60	0.58	0.56	0.56
**NOEKEON**	0.44	0.42	0.39	0.39

**Table 8:** TCP, LWC pairs ranked and clustered by score value.

LWC	TCP	*S*	Cluster
PRINCE	A3coverage	0.68	1
PRINCE	A3	0.66	1
PRINCE	EECDS	0.63	1
PRINCE	CDS rule K	0.63	1
LED	A3coverage	0.61	2
AES	A3coverage	0.61	2
PRESENT	A3coverage	0.60	2
LED	A3	0.59	2
AES	A3	0.59	2
PRESENT	A3	0.58	2
LED	EECDS	0.56	2
AES	EECDS	0.56	2
LED	CDS rule K	0.56	2
AES	CDS rule K	0.56	2
PRESENT	EECDS	0.56	2
PRESENT	CDS rule K	0.56	2
NOEKEON	A3coverage	0.44	3
NOEKEON	A3	0.42	3

**Table 9:** HERMEES scoring results.

LWC	TCP	*S* Score
PRINCE	A3coverage	0.68
PRINCE	A3	0.66
PRINCE	EECDS	0.63
PRINCE	CDS rule K	0.63

**Table 10:** Estimated energy consumption per regular node in the simulated WSN, in mJ.

	A3	A3coverage	CDS Rule K	EECDS
**AES**	4005	7585	4973	6582
**PRESENT**	11,771	22,294	14,615	19,346
**NOEKEON**	79,185	149,971	98,316	130,136
**LED**	2263	4287	2810	3720
**PRINCE**	618	1171	767	1016

## Data Availability

The original contributions presented in the study are included in the article, further inquiries can be directed to the corresponding author.

## Abbreviations

The following abbreviations are used in this manuscript:

HERMEES	Holistic Evaluation and Ranking Model for Energy Efficient Systems
MCDM	multi-criteria decision-making
LWC	lightweight cryptography
TCP	topology construction protocol
WSN	wireless sensor network
SAW	Simple Additive Weighting
IoT	Internet of Things
SPN	Substitution Permutation Network
FN	Feistel Network
IIoT	Industrial Internet of Things

## References

[1] Pandey S., Bhushan B. (2024). Recent Lightweight cryptography (LWC) based security advances for resource-constrained IoT networks. Wirel. Netw..

[2] Prvulović P., Radosavljević N., Babić Đ. Analysis of Lightweight Cryptographic Protocols in Precision Agriculture—A Case Study. Proceedings of the 2021 15th International Conference on Advanced Technologies, Systems and Services in Telecommunications—TELSIKS.

[3] Prvulović P., Radosavljević N., Babić Đ. (2021). An Overview of Lightweight Block Cipher Algorithms Based on SPN Network From the Aspect of Security of Wireless Sensor Networks. J. Mechatron. Autom. Identif. Technol..

[4] Prvulović P., Radosavljević N., Babić Đ. Pregled lakih blok-šifarskih algoritama zasnovanih na SPN mreži sa aspekta bezbednosti bežičnih sensorskih mreža. Proceedings of the 20th International Symposium INFOTEH-JAHORINA.

[5] Radosavljević N., Prvulović P., Vujošević D., Gavrić A. Traffic Analysis of A3 Topology Construction Protocol in Wireless Sensor Networks. the Proceedings of the 2022 21st International Symposium INFOTEH-JAHORINA.

[6] Radosavljević N., Babić Đ. (2021). Power consumption analysis model in wireless sensor network for different topology protocols and lightweight cryptographic algorithms. J. Internet Technol..

[7] Radosavljević N., Popović M., Vujošević D., Babić Đ. (2021). Optimal Selection of Lightweight Cipher Algorithm and Topology Construction Protocol in Wireless Sensor Networks. Stud. Inform. Control.

[8] Abdalgader K., Yousif J.H. (2022). Agricultural Irrigation Control using Sensor-enabled Architecture. KSII Trans. Internet Inf. Syst..

[9] Thakor V.A., Razzaque M.A., Khandaker M.R.A. (2020). Lightweight Cryptography for IoT: A State-of-the-Art. arXiv.

[10] Sivagurunathan S., Muthu Ganeshan V. (2022). Light weight cryptography (LWC) algorithms in terms of software metric for Industrial Internet of Things (IIOT). Adv. Appl. Math. Sci..

[11] Rana M., Mamun Q., Islam R. (2022). Lightweight cryptography in IoT networks: A survey. Future Gener. Comput. Syst..

[12] Radhakrishnan I., Jadon S., Honnavalli P.B. (2024). Efficiency and Security Evaluation of Lightweight Cryptographic Algorithms for Resource-Constrained IoT Devices. Sensors.

[13] Panahi P., Bayılmış C., Çavuşoğlu U., Kaçar S. (2021). Performance Evaluation of Lightweight Encryption Algorithms for IoT-Based Applications. Arab. J. Sci. Eng..

[14] Hatzivasilis G., Fysarakis K., Papaefstathiou I., Manifavas C. (2018). A review of lightweight block ciphers. J. Cryptogr. Eng..

[15] Banik S., Bogdanov A., Regazzoni F., Dunkelman O., Keliher L. (2015). Exploring Energy Efficiency of Lightweight Block Ciphers. Selected Areas in Cryptography—SAC 2015; Lecture Notes in Computer Science.

[16] Yogaraja C.A., Rani K.S.S. (2023). Key-based dynamic S-Box approach for PRESENT lightweight block cipher. KSII Trans. Internet Inf. Syst..

[17] Roy S., Roy S., Biswas A., Baishnab K.L. (2021). LCB: Light Cipher Block An Ultrafast Lightweight Block Cipher For Resource Constrained IOT Security Applications. KSII Trans. Internet Inf. Syst..

[18] Lo’ai, Tawalbeh, Alicea, M, Alsmadi, I. (2022). New and Efficient Lightweight Cryptography Algorithm for Mobile and Web Applications. Procedia Comput. Sci..

[19] Manoharan J.S. (2023). A Metaheuristic Approach Towards Enhancement of Network Lifetime in Wireless Sensor Networks. KSII Trans. Internet Inf. Syst..

[20] Mercy M.S., Mathana J.M., Jasmine J.S.L. (2021). An Energy—Efficient Optimal multi-dimensional location, Key and Trust Management Based Secure Routing Protocol for Wireless Sensor Network. KSII Trans. Internet Inf. Syst..

[21] Remesh Babu K.R., Preetha K.G., Saritha S., Rinil K.R. (2021). An Energy Efficient Intelligent Method for Sensor Node Selection to Improve the Data Reliability in Internet of Things Networks. KSII Trans. Internet Inf. Syst..

[22] Ilieva G., Yankova T. (2022). IoT System Selection as a Fuzzy Multi-Criteria Problem. Sensors.

[23] Ullah M., Nardelli P.H.J., Wolff A., Smolander K. (2020). Twenty-One Key Factors to Choose an IoT Platform: Theoretical Framework and Its Applications. IEEE Internet Things J..

[24] Vafaei N., Ribeiro R.A., Camarinha-Matos L.M. (2022). Assessing Normalization Techniques for Simple Additive Weighting Method. Procedia Comput. Sci..

[25] Comaniciu D., Meer P. (2002). Mean shift: A robust approach toward feature space analysis. IEEE Trans. Pattern Anal. Mach. Intell..

[26] Wightman P.M., Labrador M.A. Atarraya: A Simulation Tool to Teach and Research Topology Control Algorithms for Wireless Sensor Networks. Proceedings of the Second International ICST Conference on Simulation Tools and Techniques.

